# The Role of TEG Analysis in Patients with COVID-19-Associated Coagulopathy: A Systematic Review

**DOI:** 10.3390/diagnostics11020172

**Published:** 2021-01-26

**Authors:** Jan Hartmann, Alexis Ergang, Dan Mason, Joao D. Dias

**Affiliations:** Haemonetics Corporation, Boston, MA 02110, USA; alexis.ergang@haemonetics.com (A.E.); dan.mason@Haemonetics.com (D.M.); Joao.Dias@Haemonetics.com (J.D.D.)

**Keywords:** blood coagulation, coronavirus, COVID-19, fibrinolysis, thromboelastography, viscoelastic, TEG

## Abstract

Coronavirus disease 2019 (COVID-19)-associated coagulopathy (CAC), characterized by hypercoagulability and an increased risk of thrombotic complications, is an important consideration in the management of patients with COVID-19. As COVID-19 is a new disease, no standard of care for the diagnosis or management of its associated coagulopathy is yet established. Whole blood viscoelastic tests, such as thromboelastography (TEG^®^ hemostasis analyzer), analyze whole blood to provide a complete overview of the coagulation status. We conducted a systematic review of thromboelastography for management of patients with COVID-19, using MEDLINE (PubMed) and Cochrane databases. TEG^®^ parameter measurements and clinical outcomes data were extracted for analysis. Our review found 15 publications, with overall results showing thromboelastography can identify and assess a hypercoagulable state in patients with COVID-19. Furthermore, utilization of thromboelastography in this patient population was shown to predict thrombotic complications. The benefits of thromboelastography presented here, in addition to advantages compared with laboratory coagulation tests, position thromboelastography as an important opportunity for optimizing diagnosis of CAC and improving patient management in COVID-19. Given that the benefits of thromboelastography have already been demonstrated in several other clinical applications, we anticipate that clinical data from future studies in patients with COVID-19 will further elucidate the optimal use of thromboelastography in this patient population.

## 1. Introduction

First described in patients in China, the severe, acute respiratory syndrome coronavirus disease 2019 (COVID-19) has rapidly reached Europe and the US and led to a global pandemic of unprecedented proportion and disruption [1]. COVID-19 was initially described as a lung-focused SARS-like respiratory disease, with lead symptoms of cough, shortness of breath, and fever. However, it has quickly become clear that COVID-19 has a distinct pathophysiology, which has some similarities but also significant differences from other acute respiratory distress syndromes or septic entities. Reports have demonstrated that COVID-19 infection can lead to an array of organ-specific and systemic phenotypes, with patients who share etiologies presenting very differently and requiring different pathways of care [2].

One important complication of the disease that can impact patient care and management is COVID-19-associated coagulopathy (CAC), characterized by hypercoagulability and a prothrombotic state with increased risk of thrombotic events (TEs), including deep vein thrombosis, pulmonary embolism, ischemic stroke, and myocardial infarction [3]. The unique hypercoagulopathy state seen in patients with COVID-19 has been explored using both thrombin generation assays and viscoelastic hemostatic analyzers (VHA) [4]. Around 8% of patients with COVID-19 also report hemorrhagic complications associated with COVID-19, the most common being gastrointestinal bleeds [5]. Anticoagulation must therefore be weighed against the potential of increased risk of bleeding. The variation in hypercoagulability and bleeding rates seen in COVID-19 highlights the importance of a standardized assessment for bleeding in the disease, in order to both improve patient treatment and allow standardized comparison between reports and studies.

While elevated D-dimer and fibrin degradation product levels, and prolonged prothrombin (PT) and activated partial thromboplastin time (aPTT) have been observed in patients with CAC, there is as yet no assessment for coagulation in this disease population that meets the criteria for standard of care (as established by multicenter, prospective, randomized controlled trials, and years of real-world experience) [6]. Bleeding in patients with COVID-19 has also not been associated with laboratory evidence of coagulopathy, with PT and aPTT acting as poor predictors for bleeding risk [5]. Despite showing associations with poor prognosis, D-dimer and laboratory coagulation tests have not yet been validated for use in patients with COVID-19 through large-scale, multicenter randomized controlled trials, due to the relative recentness of the disease [6]. Furthermore, laboratory coagulation tests are carried out on platelet-poor plasma, ignoring other important components of coagulation such as platelets and the complexity of fibrinolysis [1]. This means they are not able to measure platelet-dependent coagulation processes, nor the mechanical and functional properties of the clot at fibrinolysis. In many cases, laboratory coagulation tests alone do not appear to be sensitive and/or clinically meaningful enough to detect coagulation and predict clinical outcomes [7], and can be variable between different demographic patient groups with unclear clinical interpretation [8]. The performance of D-dimer assays in patients with COVID-19 is highly variable, making it difficult to compare results from studies using different assays and to determine a standard cut-off threshold [9]. Therefore, measurements of conventional coagulation parameters cannot be considered standard of care for the diagnosis or management of COVID-19-associated coagulopathy.

VHA such as thromboelastography (TEG^®^ hemostasis analyzer: Haemonetics Corp., Boston, MA, USA) have been used to provide further insight and to characterize coagulopathy in patients with COVID-19, first in Italy and later in other parts of Europe, and the U.S. They analyze the viscoelastic clot characteristics, platelet function, and fibrinolysis in whole blood, giving a full picture of the patients’ coagulation status. We present findings from a systematic review focusing on the use of thromboelastography in patients with COVID-19 in order to identify and manage hypercoagulation. This review includes studies that used TEG^®^5000 or TEG^®^6s analyzers. While there are differences between the two analyzers, and the TEG^®^6s using a cartridge-based system, both measure the same parameters, and correlation of results between the two analyzers has been demonstrated [10,11].

## 2. Materials and Methods

In order to gain a clearer picture of the use of thromboelastography in COVID-19, we performed a systematic literature review of the MEDLINE (PubMed) and Cochrane databases to identify relevant literature assessing the role of thromboelastography in the management and treatment of patients with COVID-19. The predefined search string used for both databases was as follows: (“Covid-19” OR “Coronavirus” OR “sarscov2”) AND (“viscoelastic hemostatic” OR “TEG” OR “thromboelastography” OR “thrombelastography” OR “thromboelastographic” OR “Global haemostatic tests”). An additional search of Google Scholar was performed using the same search terms. We excluded articles that were not written in English, did not contain data on the use of thromboelastography, or did not include patients with COVID-19. In press abstracts or publications suggested by the authors were also included in the review. Information on study characteristics, TEG parameters, and clinical outcomes were extracted into an Excel spreadsheet (Version 2012, Microsoft Corporation, Washington, DC, USA) for each study. Included manuscripts and abstracts were assessed for quality and bias using the Scottish Intercollegiate Guidelines Network (SIGN) grading system [12]. The systemic review and manuscript write-up was performed in accordance with the Preferred Reporting Items for Systematic Reviews and Meta-Analyses (PRISMA) guidelines (Supplementary Digital Content 1) [13].

## 3. Results

### 3.1. Overview of Included Studies

**Figure 1:**
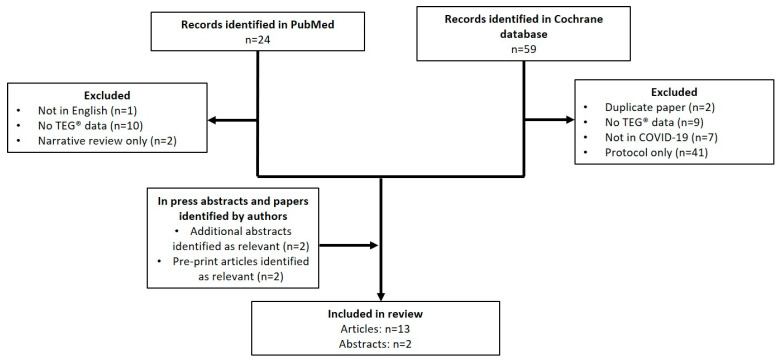
PRISMA diagram showing articles identified for inclusion in the review. COVID-19, coronavirus disease 2019; PRISMA, Preferred Reporting Items for Systematic Reviews and Meta-Analyses; TEG, thromboelastography.

In total, the PubMed search identified 24 papers and the Cochrane search identified 59 papers. Of the papers identified by the Cochrane database, 2 were duplicates, 7 did not report COVID-19 patients, 9 did not report TEG data, and the remaining 41 were trial protocols only. Of the papers identified by the PubMed search, 1 was not in English, 10 contained no thromboelastography data, and two were narrative reviews with no additional data. An additional search of Google Scholar with the same search terms revealed no further papers suitable for inclusion. In total, 11 of the manuscripts passed the predetermined inclusion criteria (Figure 1) and were included in this study. The authors suggested two additional abstracts with relevant results for inclusion, both of which were accepted for presentation at the American Heart Association meeting 14–16 November, 2020, and two preprint manuscripts currently in press.

Table 1 provides a summary of the included publications and lists the assigned SIGN gradings. Fourteen publications reported prospective or observational studies of patients with COVID-19. One case study in a single patient with COVID-19 was identified.

### 3.2. Thromboelastography Profiles in Patients with COVID-19

**Figure 2:**
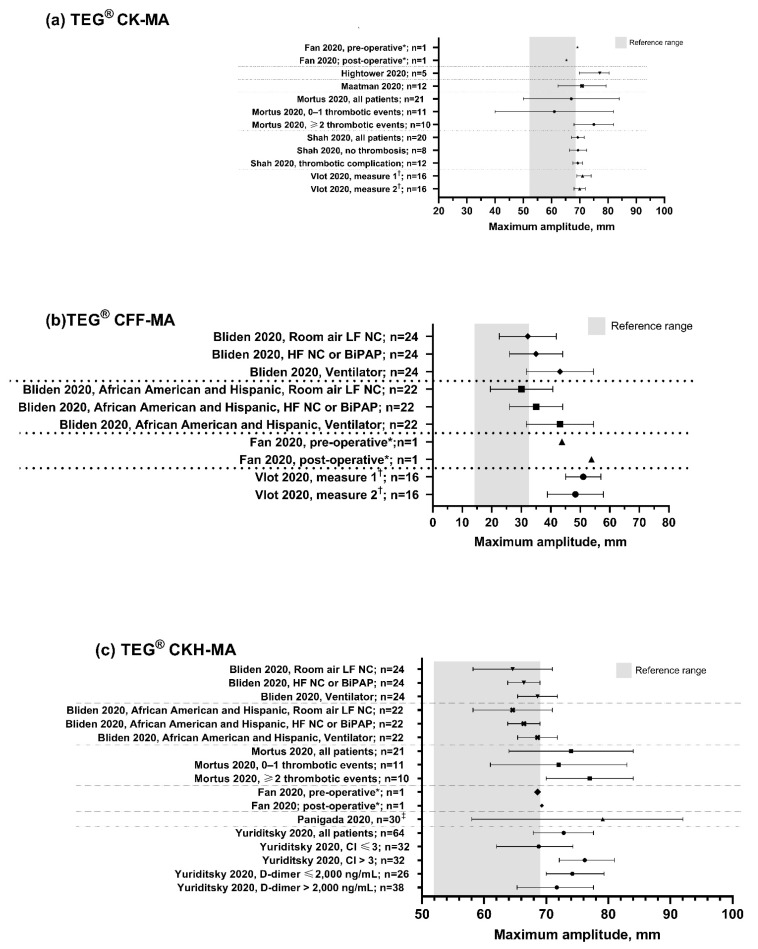
TEG MA values observed in patients with COVID-19. Grey bars show the normal TEG reference range [10]. CK, citrated kaolin assay; CFF, citrated functional fibrinogen assay; CKH, CK with heparinase assay; MA, maximum amplitude. * TEG measurements recorded before and after endovascular stent graft exclusion of the aortic thrombus and right lower limb embolectomy following diagnosis of acute ischemic limb. ^†^ Twice weekly TEG measurements recorded two weeks after the introduction of high-dose pharmacological thrombosis prophylaxis. ^‡^ Measurements were repeated on two consecutive days in six patients. Data points are mean with standard deviation error bars for normally distributed data, or median with interquartile range error bars for data that were not normally distributed. Dotted lines separate different studies with different patient populations. Data are not directly comparable between studies.

**Figure 3:**
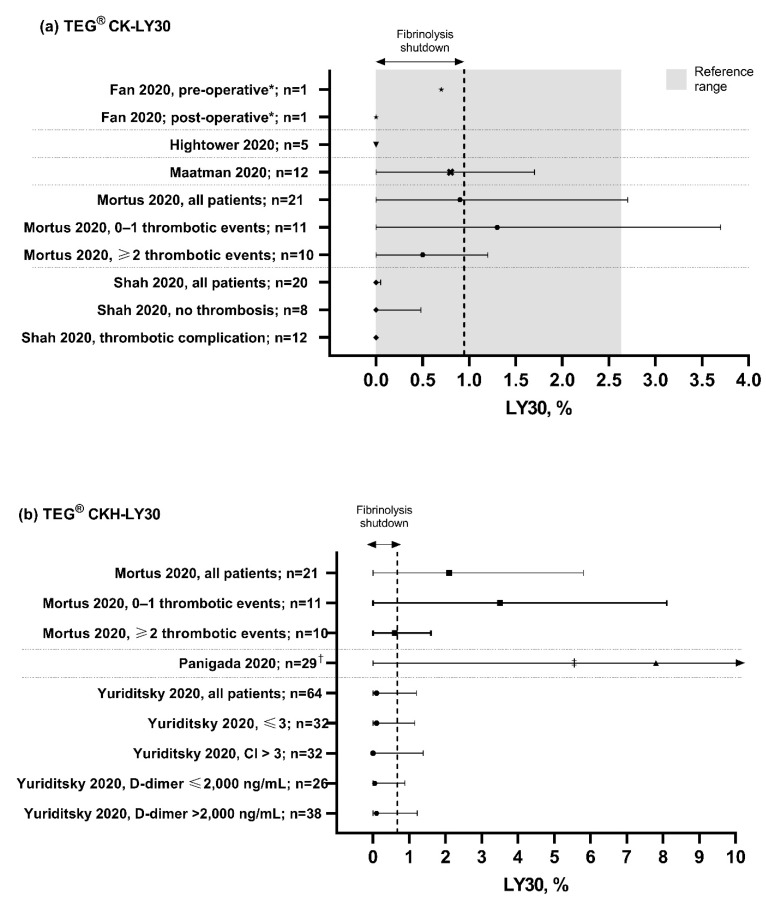
TEG LY30 values observed in patients with COVID-19. Grey bar shows the normal TEG reference range [10]. CK, citrated kaolin assay; CKH, CK with heparinase assay; LY30, clot lysis at 30 min after maximum clot strength. * TEG measurements recorded before and after endovascular stent graft exclusion of the aortic thrombus and right lower limb embolectomy following diagnosis of acute ischemic limb. ^†^ Measurements were repeated on two consecutive days in six patients. ^‡^ LY30 range reported: 0–54.3%. Data points are mean with standard deviation error bars for normally distributed data, or median with interquartile range error bars for data that were not normally distributed. Dotted lines separate different studies with different patient populations. Data are not directly comparable between studies.

**Figure 4:**
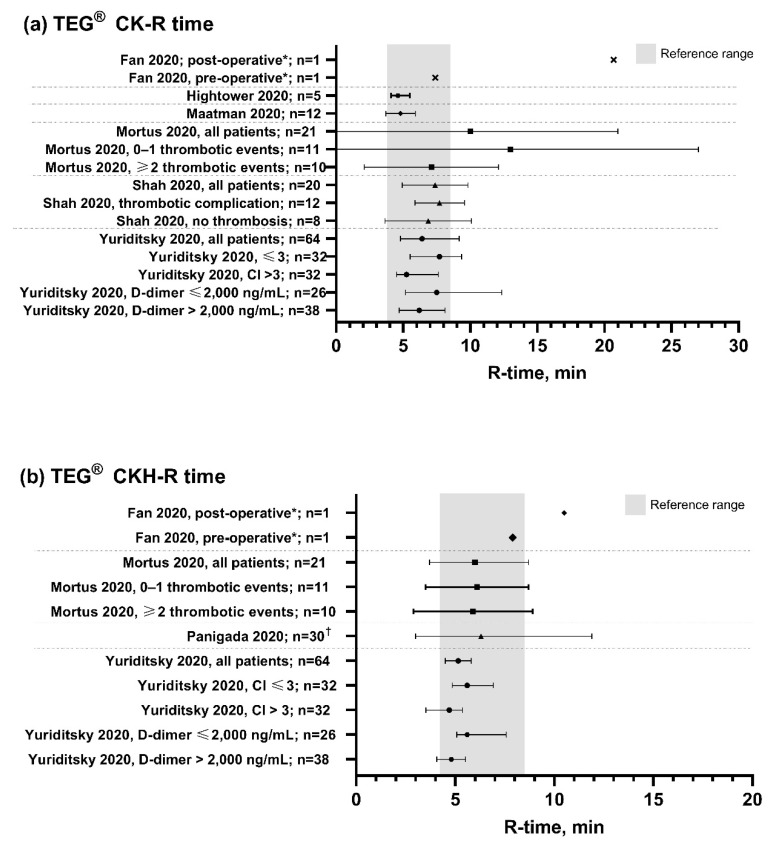
TEG R-time values observed in patients with COVID-19. Grey bars show the normal TEG reference range [10]. CK, citrated kaolin assay; CKH, CK with heparinase assay; R-time, reaction time. * TEG measurements recorded before and after endovascular stent graft exclusion of the aortic thrombus and right lower limb embolectomy following diagnosis of acute ischemic limb. ^†^ Measurements were repeated on two consecutive days in six patients. Data points are mean with standard deviation error bars for normally distributed data, or median with interquartile range error bars for data that were not normally distributed. Dotted lines separate different studies with different patient populations. Data are not directly comparable between studies.

The TEG maximum amplitude (MA), clot lysis at 30 min after maximum clot strength (LY30) and reaction time (R-time) values observed in the identified papers for patients with COVID-19 are summarized in Figure 2, Figure 3 and Figure 4, respectively.

All studies included in our review reported an increase in MA (values of MA >70 mm indicating hypercoagulability) for patients with COVID-19. Importantly, increased MA was observed in the citrated kaolin (CK)-MA channel, but also in the citrated functional fibrinogen (CFF)-MA channel—a quantifier of fibrinogen contribution to clot strength—supporting the involvement of fibrinogen in COVID-19 coagulopathy as described elsewhere [17,19,24]. One study of patients admitted to an intensive care unit (ICU) with suspicion of COVID-19 reported that elevated CFF-MA was observed in all patients with COVID-19, and that values were within normal reference ranges for all patients without COVID-19 [19]. These results were reflective of those from the other studies reporting marked increases in CFF-MA in this population [15,24], with Bliden et al. (2020) observing a stepwise increase in CFF-MA with worsening respiratory function [14,15]. Furthermore, elevations in CFF-MA reported in a single case study of a patient with COVID-19 who was ambulant while receiving treatment in a general isolation ward could suggest that quantification of fibrinogen via thromboelastography may be useful in early identification of COVID-19 coagulopathy prior to progression to severe disease [17].

Vlot et al. (2020) observed that patients with COVID-19 may also demonstrate higher fibrin contribution to clot formation relative to platelets, reporting values of median (range) 71 (56–85%) from their study [24]. Similar values for fibrin contribution to clot formation were presented in the findings of Shah et al. (2020), which also suggested that patients may show increased mean alpha angle [5].

Reduced fibrinolytic activity was also observed in patients with COVID-19, with all studies reporting LY30 less than 1%, including three which showed complete fibrinolytic shutdown (LY30 of 0%) [18,23,25]. A shortened R-time of less than 5 min was observed in patients from four studies [18,20,22,26], indicating a shorter time to clot formation in hypercoagulable patients, although the R-value was within the range of normal in most studies (Figure 4), implying that the change in R-time is less consistent than the increased MA [5,14,15,19] and fibrinolytic shutdown in the characterization of COVID-19. Platelet function, measured by the PlateletMapping ADP assay, was in the high normal range for the majority (81%) of patients with COVID-19 in the one study where this parameter was measured [15].

A summary of the values reported for thromboelastography parameters in each individual manuscript and abstract can be found in Supplementary Digital Content 2.

### 3.3. Clinical Outcomes

As well as describing the coagulation profile for patients with COVID-19, the data from the reviewed papers suggests that management of COVID-19 with thromboelastography can improve the clinical diagnosis and could potentially help in achieving better outcomes, with several presenting data to suggest that thromboelastography results may be used to predict thrombotic complications. Panigada et al. (2020) reported that increased MA corresponded to increased risk of TEs [7], while in another study, a hypercoagulable profile identified by thromboelastography was associated with a 62% TE rate [21]. Fibrinolytic shutdown, reported by TEG as a LY30 of <0.9%, correlated with thrombotic complications and mortality when combined with elevated D-dimers [20,26]. A study by Wright et al. showed that a LY30 value of 0% predicted a venous TE in 40% of patients, rising to 50% of patients when combined with D-dimer levels [25]. A LY30 value of 0% combined with elevated D-dimer levels was also found to predict the need for dialysis treatment for acute renal failure in 80% of patients [25].

Chandel et al. (2020) found that D-dimer results were less consistent for assessing hypercoagulability in patients treated with extracorporeal membrane oxygenation (ECMO), which plays a key role in COVID-19 management and treatment, reporting a significant negative correlation between thromboelastography identified hypercoagulability in patients with and without macrothrombosis [16]. This is in line with results from research that suggest that ECMO support, with TEG measurements used for ensuring ECMO maintenance, can be an integral part of critical care for patients with COVID-19 [27]. There were also data showing thromboelastography could better identify and assess hypercoagulability in patients with COVID-19 than laboratory coagulation tests such as PT and aPTT, which were found to be either normal or slightly prolonged with medians (range) 1.16 (0.99–1.50) and 0.98 (0.78–1.24), respectively [7]. In a study by Hightower et al., thromboelastography was able to identify coagulopathic states where laboratory coagulation tests could not [18]. In the study by Lawicki et al., the observation of elevated CFF-MA in all patients with COVID-19, and results within normal reference ranges for all patients without disease, was in contrast to observations for laboratory measures such as D-dimer, C-reactive protein, ferritin and procalcitonin, which were not consistently elevated in patients with COVID-19, and of which elevations were also common in patients without COVID-19 [19].

## 4. Discussion

While clinical data relating to the management of patients with COVID-19 continue to emerge at a rapid rate, the findings from this review indicate that patients display a pathognomonic hypercoagulability profile when using TEG assays. In addition, they suggest that the use of thromboelastography can lead to improvements in the clinical diagnosis of hypercoagulability and management of patients with COVID-19.

Changes in several thromboelastography parameters identify a hypercoagulable state in patients with COVID-19, namely, an increased MA, indicating high clot strength, and a low lysis time (LY30), indicating a reduction or complete shutdown in fibrinolysis. These measurements correlate with increased risk of TEs and mortality in patients with COVID-19. Some studies also report a shortened reaction time (R), although this is much less consistent. This may be a reflection of the fact that the massive inflammatory response seen in patients with COVID-19 has a greater impact on fibrinogen, von Willebrand Factor (vWF), and platelet levels, and less of an impact on coagulation factors. While the timelines of our literature search only identified one manuscript reporting the use of the TEG PlateletMapping assay to record platelet function [15], a recently published prospective study explored the use of thromboelastography with the platelet mapping algorithm to manage patients with COVID-19 [28]. The study found that the use of thromboelastography with platelet mapping could be used to characterize the spectrum of COVID-19 and reduced the risk for mechanical ventilation, acute kidney injury, and death (*p* < 0.0001 versus non-algorithm-guided patients for all). Using thromboelastography with platelet mapping to guide antiplatelet therapy treatment in patients with COVID-19 also decreased mortality by 82% (*p* = 0.0002) [28]. However, studies of platelet function using multiple electrode aggregometry did not show any change in platelet aggregability in patients with COVID-19 [29], suggesting further observations are necessary to elucidate the role of platelet function in the etiology and progression of COVID-19.

Similar hypercoagulable profiles are also seen with other VHA assays, including for the rotational thromboelastometry device ROTEM in case studies of patients with severe disease [30], and retrospective studies of patients in intensive care with severe COVID-19 [31]. Both TEG and ROTEM identify increased clot firmness in patients with COVID-19, and sometimes also report a decreased time to clot stability (although, as seen in our results, this trend is a lot weaker), with similar results seen in the Quantra hemostasis analyzer [32]. A systematic review of studies of severely ill patients with COVID-19 assessed with the TEG and ROTEM VHA found that, in line with the observations presented in this review, patients had activated blood coagulation and low fibrinolytic activity accompanied by hyperfibrinogenemia and high D-dimer levels [33]. The ClotPro VHA has also been shown to identify a hypercoagulable profile in patients with COVID-19, with increased clot strength and decreased maximum lysis, along with complete fibrinolytic shutdown observed in some patients [29,34,35]. Based on these findings, the American Society of Hematology (ASH) and American College of Surgeons (ACS) included viscoelastic tests (TEG and ROTEM) in their online COVID-19 resources [36] and recommendations on the management of coagulopathy in COVID-19 [37]. On 14 January, 2021, the U.S. FDA issued new guidance related to viscoelastic testing, such as the TEG hemostasis analyzers, during the COVID-19 pandemic. The stated intent of the policy is to “help expand the availability of coagulation systems for measurement of whole blood viscoelastic properties that are used to assess hemostasis to facilitate patient management.” [38] (https://www.fda.gov/media/145135/download).

Given the hypercoagulability observed in COVID-19, guidelines such as those from the International Society of Thrombosis and Haemostasis (ISTH) recommend treatment with heparin [39], and use of heparin adjuvants has shown improvements in clinical outcomes, including reducing patient mortality [40]. However, as bleeding has been observed in a subset of patients with COVID-19, including bleeding episodes requiring treatment with emergency lifesaving embolization, the monitoring of hemostasis is particularly important in patients receiving heparin to prevent hemorrhage [27]. In some countries, lockdowns and reduced access to medical facilities has resulted in decreased blood product availability [41], making it even more important to monitor hemostasis to prevent bleeding.

As COVID-19 is a new disease, a standard of care for the management of COVID-19-associated coagulopathy is not yet established. The clinical progression of the disease can be rapid and unpredictable; therefore, it is likely that use of whole blood assays, such as thromboelastography, offer advantages in addition to laboratory coagulation tests in terms of timely patient assessment and clinical decision making. Furthermore, evidence presented in this review suggests that thromboelastography provides an incremental value above the use of laboratory coagulation tests alone, in particular, the use of thromboelastography in addition to the data from D-dimers to predict TEs [25]. These findings are pertinent as there are some limitations of using D-dimers to monitor TEs in patients with COVID-19. Several studies in patients with COVID-19 have shown that, despite a correlation between increased D-dimer levels and a severe disease state, thrombotic events are not observed in the majority of critically ill patients with an elevated D-dimer [42]. Elevated D-dimer levels in isolation are difficult to interpret clinically as there is no standard treatment pathway based on an increased D-dimer level alone [8]. There is also the possibility that elevated D-dimer levels may be a result of mechanisms other than active lysis. To this point, the use of thromboelastography in addition to the data from D-dimers provides an indication of which results may be caused by D-dimers arising from plasmin action on crosslinked fibrin in the blood, rather than those generated through clot breakdown, and therefore indicate true fibrinolysis [43]. As COVID-19 is a relatively recent disease, one limitation of our review is that there are as yet no prospective randomized controlled trials of thromboelastography in this indication to include within a full meta-analysis. Current evidence for the utility and clinical value of thromboelastography in COVID-19 is therefore mostly from observational, retrospective studies, or from case studies of individual patients, conducted in different settings, making it more difficult to consistently extract information across the full body of literature. Papers are emerging rapidly, however, the science behind this novel disease is still being established, along with clinical norms and standards for diagnosis and treatment. A systematic review of the available evidence therefore has limited ability to make definitive therapeutic recommendations. We have also limited our review to one specific VHA device and methodology in order to analyze and compare the results of specific assays in this method-specific review.

While there is no available systematic efficacy or safety data as yet available regarding the use of TEG in patients with COVID-19, it is encouraging that no safety concerns have yet arisen. Evidence from the publications identified by the literature search demonstrate the clinical utility of thromboelastography in detecting a hypercoagulable state in patients with COVID-19, and in further providing differential diagnostic insights alongside the ability to risk-stratify patients at elevated risk for complications such as VTE or kidney failure. Given that thromboelastography has been extensively used in the critical care setting [44,45,46] and provided valuable insights into the prediction of thrombotic risk in trauma [47], oncology [48,49], and surgical patients [50], we would expect similar efficacy in the COVID-19 population. In addition, during the Ebola outbreak in 2015, thromboelastography measurements provided valuable information regarding the associated hemorrhagic coagulopathy of the novel outbreak disease [51], indicating its suitability in the case of novel diseases associated with coagulopathy.

## 5. Conclusions

This systematic literature review presents an important summary of the available clinical data on the use of thromboelastography in the management of COVID-19 coagulopathy. The evidence presented suggests that there is a consistent thromboelastography parameter profile for COVID-19-induced coagulopathy, and patient management with thromboelastography may offer opportunities to improve COVID-19 assessment and clinical outcomes. On the basis of the demonstrated benefits of thromboelastography utilization in other clinical applications, we would anticipate that, as knowledge of this relatively new disease increases, further clinical evidence, including data from prospective randomized controlled trials in this patient population, will emerge that may further elucidate the optimal use of TEG to maximize patient benefit.

## Figures and Tables

**Table 1:** Overview of included studies.

Author and Year	Population	Study Type	SIGN Grade [12] *
Bliden et al., 2020 [14]	African American and Hispanic patients hospitalized with COVID-19; n = 22	Abstract: prospective study	2−
Bliden et al., 2020 [15]	Patients hospitalized with COVID-19; n = 24	Abstract: prospective study	2−
Chandel et al., 2020 [16]	Critically ill patients with COVID-19 receiving ECMO therapy; n = 24	Retrospective study	2+
Fan et al., 2020 [17]	COVID-19 pneumonia; n = 1	Case study	3
Hightower et al., 2020 [18]	Patients with COVID-19 admitted to ICU for hypoxemic respiratory failure; n = 5	Observational study	3
Lawicki et al., 2020 [19]	ICU patients with suspected diagnosis of COVID-19; n = not stated	Retrospective study	2−
Maatman et al., 2020 [20]	Critically ill patients with COVID-19 admitted to ICU; n = 109	Observational cohort study	2+
Mortus et al., 2020 [21]	Patients with COVID-19 admitted to ICU; n = 21	Observational cohort study	2−
Panigada et al., 2020 [7]	Patients with COVID-19 admitted to ICU; n = 24	Observational study	3
Sadd et al., 2020 [22]	Patients with COVID-19 complicated by acute respiratory distress syndrome; n = 10	Retrospective observational cohort study	2−
Shah et al., 2020 [5]	Critically ill patients with COVID-19 admitted to ICU; n = 187 ^†^	Retrospective observational study	2−
Stattin et al., 2020 [23]	Critically ill patients with COVID-19 admitted to ICU; n = 31	Prospective observational study	2−
Vlot et al., 2020 [24]	Patients with COVID-19 admitted to ICU; n = 16	Observational study	3
Wright et al., 2020 [25]	Patients with COVID-19 admitted to ICU; n = 44	Observational cohort study	2−
Yuriditsky et al., 2020 [26]	Patients with COVID-19 admitted to ICU; n = 64	Retrospective cohort study	2−

* SIGN (Scottish Intercollegiate Guidelines Network) Grades: 1++ = RCTs with a very low risk of bias; 1+ = RCTs with a low risk of bias; 1− = RCTs with a high risk of bias; 2++ = High-quality case-control or cohort studies with a very low risk of confounding or bias and a high probability that the relationship is causal; 2+ = Well-conducted case-control or cohort studies with a low risk of confounding or bias and a moderate probability that the relationship is causal; 2− = Case-control or cohort studies with a high risk of confounding or bias and a significant risk that the relationship is not causal; 3 = Nonanalytic studies; 4 = Expert opinion. ^†^ TEG measurements recorded for 20 patients. COVID-19, coronavirus disease 2019; ICU, intensive care unit.

## Supplementary Materials

The following are available online at https://www.mdpi.com/2075-4418/11/2/172/s1, Table S1: PRISMA checklist; Table S2: TEG^®^ values observed in patients with COVID-19.
