# Supplementary material for: The Role of TEG Analysis in Patients with COVID-19-Associated Coagulopathy: A Systematic Review
Source: Diagnostics (Basel). 2021 Jan 26;11(2):172. doi: 10.3390/diagnostics11020172 (PMC7911186; doi:10.3390/diagnostics11020172)
Supplement: Supplementary file 1 [file diagnostics-11-00172-s001.pdf]

The Role of TEG® Analysis in Patients with COVID-19-Associated Coagulopathy: A Systematic Review

Jan Hartmann, Alexis Ergang, Dan Mason and Joao D. Dias

Supplementary Materials

Table S1. PRISMA checklist.

Section/topic	#	Checklist item	Reported on page #
**TITLE**			
Title	1	Identify the report as a systematic review, meta-analysis, or both.	1
**ABSTRACT**			
Structured summary	2	Provide a structured summary including, as applicable, background; objectives; data sources; study eligibility criteria, participants, and interventions; study appraisal and synthesis methods; results; limitations; conclusions and implications of key findings; systematic review registration number.	1
**INTRODUCTION**			
Rationale	3	Describe the rationale for the review in the context of what is already known.	1-2
Objectives	4	Provide an explicit statement of questions being addressed with reference to participants, interventions, comparisons, outcomes, and study design (PICOS).	2
**METHODS**			
Protocol and registration	5	Indicate if a review protocol exists, if and where it can be accessed (e.g., Web address), and, if available, provide registration information including registration number.	N/A
Eligibility criteria	6	Specify study characteristics (e.g., PICOS, length of follow-up) and report characteristics (e.g., years considered, language, publication status) used as criteria for eligibility, giving rationale.	2
Information sources	7	Describe all information sources (e.g., databases with dates of coverage, contact with study authors to identify additional studies) in the search and date last searched.	2
Search	8	Present full electronic search strategy for at least one database, including any limits used, such that it could be repeated.	2
Study selection	9	State the process for selecting studies (i.e., screening, eligibility, included in systematic review, and, if applicable, included in the meta-analysis).	2

Data collection process	10	Describe method of data extraction from reports (e.g., piloted forms, independently, in duplicate) and any processes for obtaining and confirming data from investigators.	2
Data items	11	List and define all variables for which data were sought (e.g., PICOS, funding sources) and any assumptions and simplifications made.	N/A
Risk of bias in individual studies	12	Describe methods used for assessing risk of bias of individual studies (including specification of whether this was done at the study or outcome level), and how this information is to be used in any data synthesis.	2, Table 1
Summary measures	13	State the principal summary measures (e.g., risk ratio, difference in means).	N/A
Synthesis of results	14	Describe the methods of handling data and combining results of studies, if done, including measures of consistency (e.g., I^2) for each meta-analysis.	N/A
Risk of bias across studies	15	Specify any assessment of risk of bias that may affect the cumulative evidence (e.g., publication bias, selective reporting within studies).	2
Additional analyses	16	Describe methods of additional analyses (e.g., sensitivity or subgroup analyses, meta-regression), if done, indicating which were prespecified.	N/A
**RESULTS**			
Study selection	17	Give numbers of studies screened, assessed for eligibility, and included in the review, with reasons for exclusions at each stage, ideally with a flow diagram.	2,3, Figure 1
Study characteristics	18	For each study, present characteristics for which data were extracted (e.g., study size, PICOS, follow-up period) and provide the citations.	3, Table 1, Appendix Table 1
Risk of bias within studies	19	Present data on risk of bias of each study and, if available, any outcome-level assessment (see item 12).	Table 1
Results of individual studies	20	For all outcomes considered (benefits or harms), present, for each study: (a) simple summary data for each intervention group (b) effect estimates and confidence intervals, ideally with a forest plot.	N/A
Synthesis of results	21	Present results of each meta-analysis done, including confidence intervals and measures of consistency.	N/A
Risk of bias across studies	22	Present results of any assessment of risk of bias across studies (see Item 15).	N/A
Additional analysis	23	Give results of additional analyses, if done (e.g., sensitivity or subgroup analyses, meta-regression [see Item 16]).	N/A
**DISCUSSION**			
Summary of evidence	24	Summarize the main findings including the strength of evidence for each main outcome; consider their relevance to key groups (e.g., healthcare providers, users, and policymakers).	9
Limitations	25	Discuss limitations at study and outcome level (e.g., risk of bias), and at review level (e.g., incomplete retrieval of identified research, reporting bias).	10
Conclusions	26	Provide a general interpretation of the results in the context of other evidence, and implications for future research.	9–10
**FUNDING**			

Funding	27	Describe sources of funding for the systematic review and other support (e.g., supply of data); role of funders for the systematic review.	11

PRISMA, Preferred Reporting Items for Systematic Reviews and Meta-Analyses

Table S2: TEG® values observed in patients with COVID-19.

Author and year	Type of study	Patient population	TEG® device	TEG® assay measurements			Clinical outcomes
				MA	LY30	R-time	
Bliden et al. AHA presentation scheduled for Nov 14–16, 2020, AHA Meeting [14]	Abstract: prospective study	African American and Hispanic patients hospitalized with COVID-19; n = 22	TEG®6s	<u>CFF-MA</u><br>COVID-19-negative patients: 15.5 ± 4.9 mm<br>Room air/LF NC patients: 30.1 ± 10.6 mm<br>HC NC/BiPAP patients: 35.0 ± 9.0 mm<br>Ventilator patients: 43.1 ± 11.4 mm; $p <$ 0.05 vs. room air/LF NC<br><br><u>CKH-MA</u><br>COVID-19 negative patients: 63.5 ± 4.4 mm<br>Room air/LF NC patients: 64.6 ± 6.4 mm<br>HC NC/BiPAP patients: 66.4 ± 2.6 mm<br>Ventilator patients: 68.6 ± 3.2 mm	COVID-19-negative patients: 0.9 ± 0.9%<br>Room air/LF NC patients: 0.3 ± 0.5%<br>HC NC/BiPAP patients: 1.3 ± 0.9%<br>Ventilator patients: 0.6 ± 1.0%	COVID-19-negative patients: 7.6 ± 3.3 min<br>Room air/LF NC patients: 6.6 ± 1.7 min<br>HC NC/BiPAP patients: 5.3 ± 0.9 min<br>Ventilator patients: 5.6 ± 1.1 min	

Author and year	Type of study	Patient population	TEG® device	TEG® assay measurements MA	LY30	R-time	Clinical outcomes
Bliden et al. AHA presentation scheduled for Nov 14–16, 2020, AHA Meeting [15]	Abstract: prospective study	Patients hospitalized with COVID-19; n = 24	TEG® 6s	CFF-MA<br>Room air/LF NC patients: 32.2 ± 9.7 mm<br>HC NC/BiPAP patients: 35.0 ± 9.0 mm<br>Ventilator patients: 43.1 ± 11.4 mm; $p < 0.05$ vs. room air/LF NC<br><br>CKH-MA<br>Room air/LF NC patients: 64.6 ± 6.4 mm<br>HC NC/BiPAP patients: 66.4 ± 2.6 mm<br>Ventilator patients: 68.6 ± 3.2 mm<br><br>ADP-MA<br>Room air/LF NC patients: 62 ± 4.7 mm<br>HC NC/BiPAP patients: 67.8 ± 1.2 mm<br>Ventilator patients: 54 ± 13 mm	Room air/LF NC patients: 0.3 ± 0.5%<br>HC NC/BiPAP patients: 1.3 ± 0.9%<br>Ventilator patients: 0.6 ± 1.0%	Room air/LF NC patients: 6.3 ± 1.7 min<br>HC NC/BiPAP patients: 5.3 ± 0.9 min<br>Ventilator patients: 5.6 ± 1.1 min	
Chandel et al. In Press [16]	Retrospective study	Critically ill COVID-19 patients receiving ECMO therapy; n = 24	TEG® 5000	CK-MA median (IQR)<br>All patients: 72.8 (71.2, 78.4) mm<br>Macrothrombosis: 74.9 (72.3, 79.9)<br>No thrombosis: 72.1 (70.6, 77.9)		R-time median (IQR)<br>All patients: 10.4 (8.5, 12.8) min<br>Macrothrombosis: 11.6 (8.6, 12.5) min<br>No thrombosis: 10.3 (8.4, 13.1) min	MA ≥68 mm associated with lower absolute D-dimer values and higher absolute fibrinogen values ($p < 0.001$) in this patient population

Author and year	Type of study	Patient population	TEG® device	TEG® assay measurements			Clinical outcomes
				MA	LY30	R-time	
Fan BE, et al. *J Thromb Thrombolysis* 2020;50:292-297 [17]	Case study	COVID-19 pneumonia; n = 1	TEG® 6s	Pre-operatively<br>CRT-MA: 71.3 mm (52–70 mm);<br>CK-MA: 69.2 mm (52–69 mm);<br>CFF-MA: 43.8 mm (15–32 mm)<br>Postoperatively<br>CKH-MA: 69.3 mm (52–69 mm)		Postoperatively<br>CKH-R prolonged relative to CK-R (20.7 vs. 10.5 min)	TEG® detected hypercoagulability even in the presence of heparin—heparin effect was detected even though measured anti-Xa activity was subtherapeutic.
Hightower, et al. *Thromb Res*. 2020;195:69-71 [18]	Observational study	Patients with COVID-19 admitted to ICU for hypoxemic respiratory failure; n = 5	TEG® 5000	CK-MA: 69.8 to 80.4 mm	0% in all patients	4.1 to 5.5 min	Analysis of the TEG® results from this small cohort highly suggest dysregulation of the fibrinolytic system as a prominent factor in promoting the hypercoagulable state observed in patients with COVID-19.
Lawicki et al In Press [19]	Retrospective study	ICU patients with suspected diagnosis of COVID-19; n = not stated	TEG® 6s	CFF-MA<br>Patients with COVID-19: elevated in 100%<br>Non-COVID-19 patients: normal in 100%<br><br>rTEG® CK-MA:<br>Patients with COVID-19: elevated in 90.5%, normal in 9.5%<br>Non-COVID-19 patients: normal in 100%		Heparinized<br>Patients with COVID-19: elevated in 90.9%, normal in 9.1%<br>Non-COVID-19 patients: elevated in 100%<br><br>Non-heparinized<br>COVID-19 patients: elevated in 20%, normal in 70%, decreased in 10%<br>Non-COVID-19 patients: normal in 75%, decreased in 25%	CFF-MA was consistently elevated in patients withCOVID-19 while normal in all patients found to be negative. D-dimer was commonly but not consistently elevated.<br>While all COVID-19-negative patients showed normal TEG® results, some had elevated levels of D-dimer and other inflammation markers.

Author and year	Type of study	Patient population	TEG® device	TEG® assay measurements MA	LY30	R-time	Clinical outcomes
Maatman TK, et al. *Crit Care Med*. 2020;48(9):e783-e790 [20]	Observational study	Critically ill patients with COVID-19 admitted to ICU; n = 109	TEG® 5000	CK-MA: Mean: 70.8 ± 8.5 mm 5/12 (42%) hypercoagulable	CK-LY30: Mean: 0.8 ± 0.9%	CK-R: Mean: 4.8 ± 1.1 mm 8/12 (67%) hypercoagulable	D-dimer and peak D-dimer were associated with VTE ($p < 0.05$). As TEG® was only performed in 12 patients, of whom only 4 developed VTE, a statistical correlation cannot be determined; however, 58% of TEG® patients had at least two hypercoagulable parameters and 83% had one.
Mortus JR, et al. *JAMA Netw Open*. 2020;3(6):e2011192 [21]	Observational cohort study	Patients with COVID-19 admitted to ICU; n = 21	TEG® 5000	Low event rate (0–1 TEs)<br>CK-MA: 61 ± 21 mm<br>CKH-MA: 72 ± 11 mm<br>High event rate (≥2 TEs)<br>CK-MA: 75 ± 7 mm<br>CKH-MA: 77 ± 7 mm	Low event rate<br>CK-LY30: 1.3 ± 2.4%<br>CKH-LY30: 3.5 ± 4.6%<br>High event rate<br>CK-LY30: 0.5 ± 0.7 mm<br>CKH-LY30: 0.6 ± 1 mm	Low event rate<br>CK-R: 13 ± 14 min<br>CKH-R: 6.1 ± 2.6 min<br>High event rate<br>CK-R: 7.5 ± 5 min<br>CKH-R: 5.9 ± 3 mm	Elevated innate MA predicted high rate of TEs (≥2 TEs); $p = 0.01$ Innate TEG® MA provided 100% sensitivity and 100% negative predictive value for TEs.
Panigada M, et al. *J Thromb Haemost*. 2020;18(7):1738-1742 [7]	Observational study	Patients with COVID-19 admitted to ICU; n = 24	TEG® 5000	CKH-MA: 79.1 (58.0–92.0) mm	CKH-LY30: 7.8 (0–54.3) mm	CKH-R: 6.3 (3.0–11.9) min	Patients with COVID-19 may develop a state of hypercoagulability as shown by the TEG® parameters.
Sadd, et al. *Crit Care Explor*. 2020;2(9):e0192 [22]	Retrospective observational cohort study	Patients with COVID-19 complicated by acute respiratory distress syndrome; n = 10	TEG® 5000	CK/CKH-MA<br>Median: 71.95 (68.5–74.5) mm	CK/CKH-LY30<br>Median: 0.75% (0–2.6%)	CK/CKH-R<br>Median: 4.45 (3.6–5.8) min	Patients who received thrombolytic therapy demonstrated improvements in coagulation index and LY30

Author and year	Type of study	Patient population	TEG® device	TEG® assay measurements			Clinical outcomes
				MA	LY30	R-time	
Shah, et al. *Crit Care.* 2020;24(1):561 [5]	Retrospective observational study	Critically ill patients with COVID-19 admitted to ICU; n = 187 (TEG®measurements recorded for 20 patients)	TEG® 6s	CK-MA<br>All patients: 69.3 (2.26)<br>With thromboembolic complications: 69.3 (1.70)<br>Without thromboembolic complications: 69.4 (3.06)	CK-LY30<br>All patients: 0.00 (0.00–0.05)<br>With thromboembolic complications: 0.00 (0.00–0.00)<br>Without thromboembolic complications: 0.00 (0.00–0.48)	CK-R<br>All patients: 7.37 (2.45)<br>With thromboembolic complications: 7.70 (1.87)<br>Without thromboembolic complications: 6.86 (3.22)	No significant differences observed between patient groups for TEG® assay results, however data were only available for 20 patients

Author and year	Type of study	Patient population	TEG® device	TEG® assay measurements			Clinical outcomes
				MA	LY30	R-time	
Stattin et al. *J Crit Care*. 2020;60:249-252 [23]	Prospective study	Critically ill patients with COVID-19 admitted to ICU; n = 31	TEG® 6s	<u>CK/CKH-MA:</u><br>All patients had MA >65 mm, while 74% patients had MA >69 mm and 42% patients had MA >72 mm at some point during ICU stay	<u>CK/CKH-LY30:</u> 0% in all patients	<u>At ICU admission</u><br>CK-R: Patients without TE = 7.2 (IQR 6.4–8.2) s; patients with TE = 6.2 (IQR 5.3–7.7) s<br>CKH-R: Patients without TE = 7.0 (IQR 6.2–7.7) s; patients with TE = 6.5 (IQR 5.4–8.5) s<br><br><u>Day <4 (all patients)</u><br>CK-R: 7.3 (IQR 6.7–8.2) s<br>CKH-R: 6.9 (IQR 6.2–7.7) s<br><br><u>Day 4–7 (all patients)</u><br>CK-R: 8.9 (IQR 7.3–10.6) s<br>CKH-R: 8.3 (IQR 6.8–9.4) s<br><br><u>Day >7 (all patients)</u><br>CK-R: 8.1 (IQR 6.1–9.5) s<br>CKH-R: 7.6 (IQR 5.7–10.1) s	Patients with COVID-19 have hypercoagulability with high MA on TEG®.

Author and year	Type of study	Patient population	TEG® device	TEG® assay measurements			Clinical outcomes
				MA	LY30	R-time	
Vlot et al. *Thromb Res*. 2020;196:1-3 [24]	Observational study	Patients with COVID-19 admitted to ICU; n = 16	TEG® 6s	<u>CK-MA</u>: 71 (IQR 69–74) mm at time point 1 and 70 (IQR 68–72) mm at time point 2<br><u>CFF-MA</u>: 51 (IQR 45–57) mm at time point 1 and 48 (IQR 39–58) mm at time point 2			TEG® MA assays demonstrated a procoagulant pattern.
Wright et al. *J Am Coll Surg*. 2020;231(2):193-203.e1 [25]	Observational study	Patients with COVID-19 admitted to ICU; n = 44	TEG® 5000	<u>CK/CKH-MA</u><br>All patients: 73 (IQR 67–77) mm<br>73 (IQR 66–78) mm in patients with complete fibrinolysis shutdown and 77 (IQR 72–78) mm in patients without complete fibrinolysis shutdown; *p* = 0.999	<u>CK/CKH-LY30</u>:<br>0 (IQR 1–0.4) %<br><br>0% in 57% of patients	<u>CK/CKH-R</u><br>All patients: 5.8 (IQR 4.8–8.6) min<br>6 (IQR 4.5–10.2) min in patients with complete fibrinolysis shutdown and 7.1 (IQR 4.5–7.8) min in patients without complete fibrinolysis shutdown; *p* = 0.537	Fibrinolysis shutdown, evidenced complete failure of LY30 on TEG®, predicts TEs and the need for hemodialysis in critically ill patients with COVID-19.
Yuriditsky et al. *Crit Care Med.* 2020;48:1319-1326 [26]	Retrospective study	Patients with COVID-19 admitted to ICU; n = 64	TEG® 5000	<u>CKH-MA</u> in the hypercoagulable range (70 mm) in 60.1% of patients	<u>CK-LY30</u> within normal range (0–8%) in 95.3% of patients	<u>CK-R</u> below normal (<5 min) in 43.8% of patients	While TEG® did not distinguish between patients with higher or lower D-dimers or VTE, the hypercoagulable TEG® profiles showed a significant contribution of fibrinogen and platelets to the hypercoagulability.

ADP = adenosine diphosphate; BiPAP = bilevel positive airway pressure; CFF = citrated functional fibrinogen; CK = citrated kaolin; CKH = citrated kaolin with heparinase; COVID-19 = coronavirus disease 2019 ; CRT = citrated rapid TEG; HF NC = high-flow nasal cannula; ICU = intensive care unit; IQR = interquartile range; LF NC = low-flow nasal cannula; LY30 = amplitude at 30 min; MA = maximum amplitude; R = reaction time; TE = thrombotic events; TEG®= thromboelastography; VTE = venous thromboembolism
